# Patch-Clamp Analysis of the Mitochondrial H^+^ Leak in Brown and Beige Fat

**DOI:** 10.3389/fphys.2020.00326

**Published:** 2020-04-15

**Authors:** Ambre M. Bertholet, Yuriy Kirichok

**Affiliations:** Department of Physiology, University of California, San Francisco, San Francisco, CA, United States

**Keywords:** mitochondria, H^+^ leak pathway, uncoupling, thermogenesis, brown fat, beige fat, patch-clamp technique, fatty acid

## Abstract

Mitochondria convert the chemical energy of metabolic substrates into adenosine triphosphate (ATP) and heat. Although ATP production has become a focal point of research in bioenergetics, mitochondrial thermogenesis is also crucial for energy metabolism. Mitochondria generate heat due to H^+^ leak across the inner mitochondrial membrane (IMM) which is mediated by mitochondrial uncoupling proteins. The mitochondrial H^+^ leak was first identified, and studied for many decades, using mitochondrial respiration technique. Unfortunately, this method measures H^+^ leak indirectly, and its precision is insufficient for the rigorous insight into the mitochondrial function at the molecular level. Direct patch-clamp recording of H^+^ leak would have a significantly higher amplitude and time resolution, but application of the patch-clamp technique to a small subcellular organelle such as mitochondria has been challenging. We developed a method that facilitates patch-clamp recording from the whole IMM, enabling the direct measurement of small H^+^ leak currents via uncoupling proteins and thus, providing a rigorous understanding of the molecular mechanisms involved. In this paper we cover the methodology of measuring the H^+^ leak in mitochondria of specialized thermogenic tissues brown and beige fat.

## Introduction

The electrical potential and ion transport across the inner mitochondrial membrane (IMM) are central for mitochondrial physiology. The patch-clamp technique revolutionized our understanding of electrical signaling at the cell plasma membrane, leading to the development of important drugs that control the activity of cardiac and neuronal ion channels. However, similar advanced application of the patch-clamp electrophysiology to mitochondria was considered impossible because of their smaller size and intricate morphology.

Only single-channel recording from small membrane patches of the IMM, either in the “membrane-attached” mode or in “inside-out” mode, were routinely used. In contrast, patch-clamp recording from the whole IMM (the whole-IMM mode), which is analogous to whole-cell recording from the plasma membrane, was considered prohibitively difficult. Thus, direct studies of the mitochondrial transport proteins were limited to relatively abundant, large-conductance ion channels that are encountered often and resolved well in the “membrane-attached” or “inside-out” modes. Unfortunately, such ion channels represent only a small fraction of the mitochondrial transport proteins. Even recording and analyzing unitary currents of smaller ion channels such as mitochondrial Ca^2+^ uniporter (MCU, single-channel conductance ∼5 pS), were difficult and necessitates using supraphysiological concentrations of permeable ions and sylgard-covered pipettes to improve signal-to-noise ratio (Leech and Holz, 1994). Patch-clamp studies of carriers, which perhaps represent the major fraction of all IMM transport proteins, are not possible at all. This is because their transport rates are orders of magnitude slower as compared to channels, and unitary currents cannot be resolved.

An additional, and often overlooked, complication associated with single-channel recording in the membrane-attached or inside-out modes is a very high membrane tension present in small membrane patches under the patch pipette. This tension can be as high as 10–40% of the force required for membrane lysis (Suchyna et al., 2009). Such tension is absent in the whole-cell (or whole-IMM) mode, and an ion activity that requires tension can sometimes be recorded only in the membrane-attached/inside-out modes (Zhang and Hamill, 2000). Because of the unusually high tension, the electrophysiological data obtained from small membrane patches must be treated with caution. Specifically, the ion channels observed may not be active under regular physiological conditions but activated only in certain special/pathological states. Because of its applicability to a large number of transport proteins, better control over membrane tension, and simple analysis of macroscopic currents, the whole-cell patch-clamp has been the dominant method to study plasma membrane ion channels. The membrane-attached or inside-out modes single-channel analysis has been normally used to provide additional mechanistic insight. Therefore, the inability to study mitochondrial ion channels and transporters in the whole-IMM mode was a major technical barrier in bioenergetics.

This barrier has now been eliminated with the development of a method for reproducible patch-clamp recording from the whole native IMM, allowing high-resolution functional analysis of a much larger fraction of mitochondrial ion channels and transporters in their native membrane environment (Kirichok et al., 2004; Fedorenko et al., 2012; Fieni et al., 2012; Bertholet et al., 2017, 2019; Garg and Kirichok, 2019). This method can be applied to mitochondria isolated from different tissues, such as skeletal muscle, heart, kidney, liver, and brown and beige adipose tissues. This technique opens new possibilities for the identification and characterization of the molecular mechanisms underlying the conversion of energy within mitochondria.

The patch-clamp electrophysiology is often equated with the art of formation of the gigaohm seal between the glass patch pipette and the membrane. Although this step is also essential in the whole-IMM patch-clamp, it is far from being sufficient. Gigaom seals between the glass patch pipette and the vesicles of the whole IMM (mitoplasts) form rather easily and were reported as early as in 1987 (Sorgato et al., 1987). This, in principle, opened a possibility of both single-channel and whole-IMM recording, but only single-channels recording from mitoplast was adopted by many labs, while the whole-IMM recording was thought of as impossible. The whole-IMM mode was normally obtained after a spontaneous break-in into the mitoplast (Sorgato et al., 1987; Klitsch and Siemen, 1991), and only one paper appeared to report a controlled break-in (Borecky et al., 1997). Our experience with mitochondrial patch-clamp over years suggests that there are two important reasons for the whole-IMM patch-clamp to be challenging. First, in contrast to the cell, mitoplast has no internal cytoskeleton/organelles and can easily be destroyed by sucking it into the pipette during the break-in, unless the suction during the break-in is very gentle and used in combination with voltage. Second, if IMM integrity is compromised during mitoplast isolation, it is nearly impossible to work in the whole-IMM mode, while recording from the small membrane patches in the membrane-attached or inside-out modes could still work. Therefore, the mitoplast preparation and break-in procedure are crucial for successful whole-mitoplast recording, and our group spend significant amount of time optimizing these steps.

This manuscript focuses on the use of the whole-IMM mitochondrial patch-clamp to study the molecular mechanism of mitochondrial uncoupling in the specialized thermogenic tissues brown fat and beige fat. First, we briefly introduce the concepts of mitochondrial uncoupling and thermogenesis in their connection with the H^+^ leak across the IMM. We then discuss the specialized thermogenic tissues brown fat and beige fat as well as the molecular mechanisms by which the mitochondrial H^+^ leak is mediated by the brown/beige fat-specific uncoupling protein 1 (UCP1). Finally, we present a detailed protocol for studying the H^+^ and fatty acid (FA) anion currents mediated by UCP1 using the whole-IMM mode of the mitochondrial patch-clamp.

## Adaptive Thermogenesis, H^+^ Leak Across the Imm, and Mitochondrial Uncoupling Proteins

Mammals maintain a core body temperature of about 37°C. The heat to support this body temperature comes from various exothermic reactions. The vast majority of these reactions, grouped under the term “obligatory thermogenesis,” also perform important physiological functions other than heat production, such as protein and DNA synthesis/degradation and re-generation of ionic gradients across neuronal/cardiac membranes (Rolfe and Brown, 1997; Lowell and Spiegelman, 2000). Such reactions cannot be controlled to adjust thermogenesis and core body temperature without negatively affecting the physiology of the whole body.

Obligatory thermogenesis is sufficient to support the core body temperature when the environmental temperature is above 28°C (referred to as thermoneutrality) (Lowell and Spiegelman, 2000). However, when the ambient temperature is below thermoneutrality, engagement of an additional thermogenic mechanism, called “adaptive thermogenesis,” becomes necessary (Lowell and Spiegelman, 2000). In contrast to the obligatory thermogenesis, the exothermic reactions involved in adaptive thermogenesis occur specifically for heat production, and their thermogenic activity can be regulated to precisely control core body temperature. The specialized thermogenic tissues, brown fat and beige fat, are responsible for adaptive thermogenesis. In addition to maintaining core body temperature, adaptive thermogenesis has another important physiological role – regulating adiposity and body weight. Adaptive thermogenesis consumes large amounts of energy from fat depots and reduces their size. Given that there is a huge void in available obesity treatments, the development of pharmacological interventions that stimulate adaptive thermogenesis has the potential to transform this field.

**FIGURE 1:**
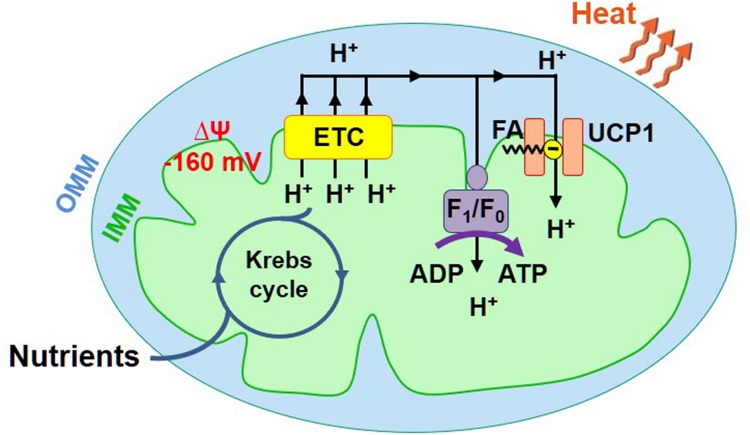
Mitochondrial ATP and heat production in brown/beige fat. Mitochondria have two membranes, the inner mitochondrial membrane (IMM) and the outer mitochondrial membrane (OMM). The IMM contains the energy conversion machinery. The Krebs cycle and electron transport chain (ETC) convert the chemical energy of nutrients into the electrical potential (ΔΨ) across the IMM, which is then converted into ATP or heat. ΔΨ (−160 mV, negative in the mitochondrial matrix) is directly generated by the ETC that pumps H^+^ across the IMM. ΔΨ is converted into ATP or heat by ATP synthase (AS) or UCP1, respectively.

Mitochondria are directly responsible for the adaptive thermogenesis mediated by brown and beige fat. Mitochondria of all tissues generate two forms of energy: ATP and heat. However, the mitochondrial ATP/heat production ratio varies significantly between different tissues. The energy output of mitochondria of the majority of tissues is, on average, about 20% heat and 80% ATP (Rolfe et al., 1999), while mitochondria of the specialized thermogenic tissue brown fat generate primarily heat (Cannon and Nedergaard, 2004). To produce ATP and heat, mitochondria use the same energy source – the voltage (ΔΨ, Figure 1) across the IMM generated by the electron transport chain (ETC). ATP is produced by the ATP synthase, while heat is produced by so-called uncoupling proteins (UCP). Both pass H^+^ down ΔΨ, but the ATP synthase uses the released energy to generate ATP from ADP and inorganic phosphate, while uncoupling proteins just let the energy dissipate as heat (Figure 1; Krauss et al., 2005). UCPs are named “uncoupling proteins” because they break coupling between the H^+^ flows via the ETC and ATP synthase. The uncoupling H^+^ current mediated by UCPs is called “H^+^ leak.” UCP1, a protein belonging to a superfamily of mitochondrial solute carriers (SLC25), is the mitochondrial UCP of the specialized thermogenic tissues brown and beige fat (Cannon and Nedergaard, 2004; Shabalina et al., 2013; Bertholet et al., 2017). UCP1 expression is limited to these two tissues, and it is required for adaptive thermogenesis (Enerback et al., 1997; Feldmann et al., 2009).

**FIGURE 2:**
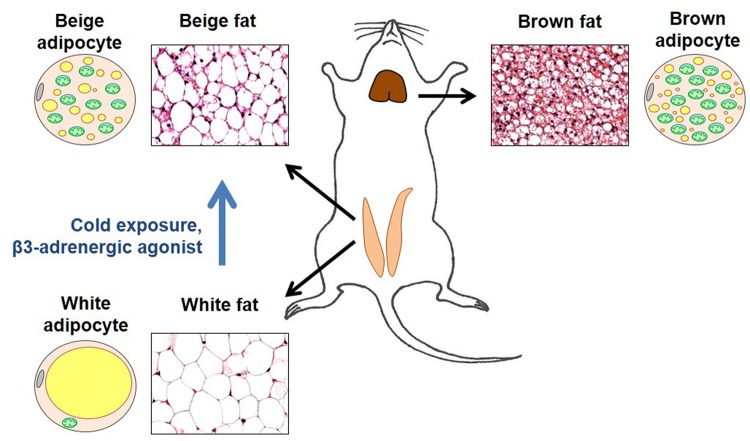
Brown and beige fat. Representative sections of the inguinal fat depot (in two states – with and without beige adipocytes) and interscapular fat depot are shown along with diagrams demonstrating morphological features of brown, white, and beige adipocytes. In the adipocyte diagrams, mitochondria are shown in green, cytoplasmic lipid droplets in yellow, and cell nucleus in gray. Brown adipocytes are characterized by a large number of mitochondria (that give this tissue its brown color) and numerous small cytoplasmic lipid droplets. In contrast, white adipocytes have a very low number of mitochondria and contain a single large lipid droplet that occupies almost all of the cytosol. In thermoneutrality, white adipocytes are the predominant type of adipocyte in the inguinal depot. However, under certain conditions such as cold exposure or injection with β3 adrenergic agonists, the inguinal depot develops beige adipocytes that are morphologically similar to brown adipocytes.

Brown fat is a specialized thermogenic tissue that makes mammals capable of protecting their core body temperature across a wide range of ambient temperatures (Lowell and Spiegelman, 2000). In contrast to white fat, brown fat is not a fat store. It accumulates fat to “burn” it (use as a fuel for adaptive thermogenesis). Brown adipocytes have numerous small cytoplasmic lipid droplets (such adipocytes are called “multilocular”), while white adipocytes contain one large lipid droplet that takes up almost the entire cytosol (Figure 2). Moreover, brown adipocytes have numerous mitochondria that express UCP1 and specialize in heat production, while white adipocytes do not express UCP1 and have a very low number of mitochondria (Figure 2).

Originally, brown fat was considered the only site of adaptive thermogenesis. However, it was later discovered that under certain conditions, such as prolonged cold exposure, increased sympathetic tone, or physical exercise, white fat develops islands of UCP1-positive adipocytes with the morphology of brown adipocytes. Specifically, similar to brown adipocytes, these adipocytes are “multilocular” and have a large number of mitochondria (Figure 2). These brown-like adipocytes are called beige (or brite) adipocytes (Kozak et al., 2010; Walden et al., 2012; Wu et al., 2012, 2013).

After identification of beige fat as a distinct type of a thermogenic fat, UCP1 was universally used as a marker of beige adipocytes, and it was generally accepted that all beige adipocytes possess the UCP1-dependent mitochondrial thermogenesis. However, direct patch-clamp characterization of H^+^ leak across the IMM of subcutaneous and abdominal beige fat demonstrated the existence of UCP1-positive and UCP1-negative beige adipocytes (Bertholet et al., 2017).

All mitochondria from subcutaneous (inguinal) beige fat, analyzed with patch-clamp electrophysiology, had UCP1-dependent H^+^ leak similar to that found in brown fat. In contrast, only about 15% of the mitochondria in abdominal (epididymal) beige fat have the UCP1-dependent H^+^ leak, with UCP1 currents being undetectable in the remaining 85% (Bertholet et al., 2017). Thus, the mitochondrial patch-clamp analysis revealed a new population of beige adipocytes that do not use UCP1 for thermogenesis (Bertholet et al., 2017). In mice, the UCP1-negative beige adipocytes are abundant in abdominal fat, whereas UCP1-positive beige adipocytes are the predominant type of beige adipocyte in subcutaneous fat.

The UCP1-negative beige adipocytes and UCP1-positive beige adipocytes share significant similarities. They are multilocular, have large mitochondrial network, robust thermogenic gene program and a distinctive OXPHOS profile different from brown fat (Bertholet et al., 2017). Moreover, the UCP1-negative and UCP1-positive beige adipocytes are both capable of heat production via a futile cycle of creatine phosphorylation-dephosphorylation (Bertholet et al., 2017; Roesler and Kazak, 2020). However, UCP1-negative beige adipocytes differ significantly from white adipocytes in thermogenic gene expression, cytoplasmic lipid droplet morphology, and mitochondrial biomass (Bertholet et al., 2017). Thus, the multilocular UCP1-negative beige cells are indubitably a distinct type of thermogenic beige adipocyte.

Despite the existence of UCP1-negative beige adipocytes, UCP1-dependent mitochondrial thermogenesis is the primary mechanism of adaptive thermogenesis. In contrast, the creatine-driven futile cycling is unlikely to be controlled by ambient temperature on an acute basis (Bertholet et al., 2017), and thus, does not contribute to the bona-fide adaptive thermogenesis. Therefore, here we focus on the mechanisms of UCP1-dependent H^+^ leak and adaptive thermogenesis.

## The Mechanism of H^+^ Leak Via Ucp1

At thermoneutrality and higher temperatures (28°C and higher), UCP1-dependent adaptive thermogenesis is suppressed. Mechanistically, under these conditions, H^+^ leak via UCP1 is inhibited by cytosolic purine nucleotides (predominantly by the Mg^2+^-free form of ATP) (Cannon and Nedergaard, 2004). When the ambient temperature drops below thermoneutrality, UCP1-dependent thermogenesis in brown/beige fat is stimulated by epinephrine released by the sympathetic nervous system. Epinephrine activates β3-adrenergic receptors, leading to hydrolysis of the cytoplasmic lipid droplets and release of free long-chain fatty acids (FA) (Cannon and Nedergaard, 2004). FA then remove purine nucleotide inhibition and activate UCP1 to increase the H^+^ conductance of the IMM and activate mitochondrial thermogenesis. However, the mechanism by which UCP1 facilitates the FA-dependent increase in H^+^ conductance remained unresolved (Cannon and Nedergaard, 2004).

Several different models of the FA- and purine nucleotide-dependent activity of UCP1 have been proposed. The principal differences between them are:

(1) Whether or not FA are required for H^+^ permeation via UCP1. Alternatively, it has been proposed the FA may simply remove purine nucleotide inhibition to unmask a constitutive H^+^ conductance of UCP1 (Cannon and Nedergaard, 2004; Shabalina et al., 2004).
(2) Whether or not H^+^ are directly translocated by UCP1. Alternatively, UCP1 was proposed to be a FA anion carrier that facilitates transmembrane FA cycling across the IMM (Garlid et al., 1996, 2000). Such UCP1-dependent FA cycling would cause FA-mediated, protonophoric H^+^ transport through the lipid bilayer (rather than direct H^+^ transport via UCP1).
(3) Whether or not FA directly compete with purine nucleotides to remove the purine nucleotide inhibition of UCP1. Alternatively, the removal of purine nucleotide inhibition by FA is non-competitive (Winkler and Klingenberg, 1994; Shabalina et al., 2004).

Direct application of patch-clamp electrophysiology to the IMM of brown and beige fat helped answer these questions and resulted in refinement of the mechanism of the FA/purine nucleotide-dependent H^+^ leak via UCP1. The following subsections summarize the most recent insights into the mechanism of UCP1 action based on electrophysiological analysis.

### FA Are Required for UCP1-Dependent H^+^ Leak

**FIGURE 3:**
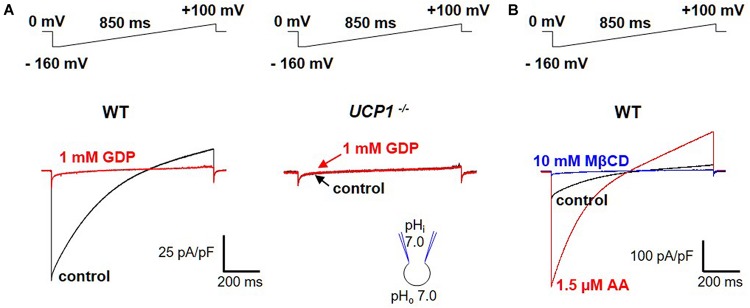
FA-dependent H^+^ currents via UCP1. **(A)** *Left panel:* the H^+^ current via UCP1 (control, black) is inhibited by 1 mM guanosine 5′-diphosphate (GDP), a classical UCP1 inhibitor (red); *Right panel:* the same experiment performed in *UCP1*^–/–^ mice; **(B)** H^+^ current via UCP1 (control, black) is de-activated by 10 mM MβCD (blue) and re-activated by 1.5 μM arachidonic acid (AA, red). The voltage protocols are indicated above the corresponding traces. Reproduced with permission from Bertholet et al. (2017).

For a transporter, UCP1 generates an unexpectedly large transmembrane current as measured across the whole IMM using the patch-clamp technique (Fedorenko et al., 2012; Bertholet et al., 2017; Figure 3A, left panel). This current is absent in *UCP1*^–/–^ mice (Figure 3A, right panel). The UCP1 current is H^+^-selective, as its reversal potentials correspond to the theoretical Nernst equilibrium potentials for H^+^ (Fedorenko et al., 2012). The H^+^ current carried by UCP1 is one of the largest H^+^ currents recorded across the native membrane. The large amplitude of the UCP1 current is especially surprising because transporters have at least three orders of magnitude lower unitary currents as compared to ion channels (Hille, 1992) and only a few of them generate currents that are large enough to be resolved with the patch-clamp technique in their native membrane. The extremely high level of UCP1 expression in brown and beige fat (Ricquier and Kader, 1976; Lin and Klingenberg, 1980; Bertholet et al., 2017) can explain the large whole-IMM H^+^ currents observed. However, as expected for a transporter, the UCP1 unitary current is too small to be resolved using the patch-clamp electrophysiology (Fedorenko et al., 2012).

Interestingly, the large UCP1-dependent H^+^ current recorded across the IMM of brown and beige fat does not seem to require FA and is recorded without application of FA (Fedorenko et al., 2012; Bertholet et al., 2017; Figure 3A, left panel). However, an “in-depth” analysis demonstrated that the H^+^ current via UCP1 is activated due to the presence of endogenous FA within the IMM of brown and beige fat (Fedorenko et al., 2012; Bertholet et al., 2017). These endogenous FA are generated via membrane phospholipid hydrolysis due to a yet unidentified phospholipase associated with the IMM (Fedorenko et al., 2012). When the endogenous FA are fully extracted from the IMM by FA acceptors cyclodextrin or albumin, the H^+^ current via UCP1 is completely de-activated (Fedorenko et al., 2012; Bertholet et al., 2017; Figure 3B). Subsequent addition of exogenous FA on the cytosolic face of the IMM reactivates the UCP1-dependent H^+^ current (Fedorenko et al., 2012; Bertholet et al., 2017; Figure 3B). Alternatively, after de-activation of UCP1 with cyclodextrin, H^+^ current via UCP1 can be recovered simply by washing out cyclodextrin from the bath solution to allow the phospholipases to re-generate endogenous membrane FA (Fedorenko et al., 2012; Bertholet et al., 2017).

These experiments clearly demonstrate that UCP1 activity indeed results in H^+^ translocation across the IMM and that FA are absolutely required for this H^+^ transport. The previous confusion regarding the “constitutive” H^+^ transport activity of UCP1 is likely due to the inability to fully extract endogenous FA generated by the IMM-associated phospholipase(s). The mitochondrial phospholipase activity profoundly affects UCP1 activity in electrophysiological experiments (Fedorenko et al., 2012; Bertholet et al., 2017) and can play a major role in the regulation of UCP1-dependent adaptive thermogenesis *in vivo*.

### H^+^/FA Anion Cotransporter as a Model for UCP1 Function

The absolute requirement of FA for the UCP1-dependent H^+^ transport (Fedorenko et al., 2012) raises the question about the mechanism by which FA could be involved. In one of the original models of UCP1 function put forward before the direct electrophysiological studies, UCP1 works as a FA anion carrier that facilitates FA cycling across the IMM. Such FA cycling leads to FA-mediated protonophoric transport of H^+^ across the lipid bilayer and not via UCP1 directly (the “FA-cycling” model) (Garlid et al., 1996, 2000). Alternatively, it was proposed that UCP1 transports H^+^ directly, and FA serve as essential co-factors that bind within UCP1 translocation pathway to enable H^+^ binding and translocation (the “H^+^-buffering” model) (Klingenberg and Huang, 1999; Klingenberg, 2017). Therefore, determination the ability of UCP1 to transport FA anions was crucial for understanding of the mechanism of UCP1-dependent H^+^ leak, and such ability was tested directly using the mitochondrial patch-clamp.

**FIGURE 4:**
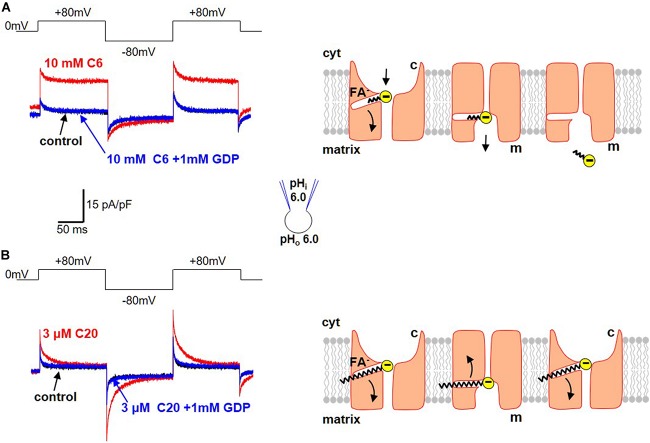
Transport of FA anions by UCP1. **(A)** Transport of short-chain alkylsulfonates by UCP1. *Left panel*: application of 10 mM C6-sulfonate (C6) on the cytosolic side of the IMM results in a steady FA anion current via UCP1 (red). This FA anion current is inhibited by the classical UCP1 inhibitor GDP (blue). The control trace (before C6 addition) is shown in black. Voltage protocol is shown above. *Right panel*: the mechanism of FA anion translocation by UCP1. UCP1 has two conformation states, with the SBS exposed either to the cytosolic (c) or matrix (m) side of the IMM. UCP1 binds a short-chain FA anion in the c-state and upon a c-m conformation change, releases the short-chain FA anion on the matrix site of the IMM. To reflect the fact that long-chain FA anions cannot bind to UCP1 on the matrix side, the access to the SBS in the m-state is shown as narrower than that in the c-state. **(B)** Limited translocation of the long-chain FA within UCP1. *Left panel*: application of 3 μM C20-sulfonate (C20) on the cytosolic side of the IMM results in a transient FA anion current via UCP1 [red, compare to **(A)**]. The transient FA anion current is inhibited by the classical UCP1 inhibitor GDP (blue). The control trace (before C20 addition) is shown in black. Voltage protocol is shown above. *Right panel*: the mechanism of transient UCP1 current induced by long-chain FA anions. A long-chain FA anion is translocated by UCP1 similar to a short-chain FA anion, but the long carbon tail of the long-chain FA anion establishes strong hydrophobic interaction with UCP1, preventing release of the FA anion from UCP1. Thus, the negatively charged FA anion shuttles within the UCP1 translocation pathway in response to the transmembrane voltage steps, producing transient currents. These currents suggest that the UCP1 SBS may slightly change its position within the membrane during the c–m conformation change. Current traces reproduced with permission from Bertholet et al. (2017).

Regular FA activate H^+^-selective transmembrane currents mediated by UCP1-dependent current (Figure 3). In striking contrast, it was determined that low-pKa FA analogs (that could not be protonated at physiological pH and existed only in the negatively charged form) instead induce FA anion currents via UCP1 (Figures 4A,B; Fedorenko et al., 2012; Bertholet et al., 2017). These experiments demonstrated that UCP1 indeed transports FA anions. Moreover, the ability of a FA to bind H^+^ at physiological pH was determined to be essential for its ability to activate H^+^ currents via UCP1.

Interestingly, a major difference was observed in the way that long-chain and short-chain FA anions interact with UCP1. First, with an increase in the length of the carbon chain (and its hydrophobicity) comes a dramatic increase in the ability of FA to induce currents via UCP1 (Fedorenko et al., 2012). Long-chain FA activate UCP1 currents in the micromolar concentration range, whereas millimolar concentrations of short-chain FA are required for the same current amplitude.

Second, long-chain and short-chain FA anions are transported by UCP1 differently. Specifically, short-chain FA anions are simply carried by UCP1 across the IMM and produce steady currents in response to steps of transmembrane voltage (Figure 4A). In contrast, long-chain FA induce transient UCP1 currents in the response to the same voltage steps (Figure 4B). These transient UCP1 currents are due to the fact that the long-chain FA anions cannot dissociate from UCP1 due to strong hydrophobic interactions, resulting in an incomplete transport cycle and only limited motion of the long-chain FA within UCP1 (Figure 4B).

Finally, long-chain FA anions bind to UCP1 and induce UCP1 currents only on the cytosolic side of IMM, while the short-chain FA anions can bind on both the cytosolic and matrix sides (Fedorenko et al., 2012). Thus, the access to the UCP1 substrate binding site from the matrix is restricted for long-chain FA due to their larger size. The inability of UCP1 to bind long-chain FA on the matrix side argued strongly against the FA-cycling model as the mechanism of FA-dependent H^+^ transport by UCP1. This was because the FA-cycling model postulated binding of long-chain FA to UCP1 on the matrix side of the IMM for H^+^ entry into mitochondria (Garlid et al., 1996, 2000). Thus, it was determined that not only H^+^ but also FA anions are UCP1 transport substrates.

The models of UCP1 operation that were proposed previously could not explain the new patch-clamp data on FA–UCP1 interaction (Fedorenko et al., 2012). The “FA-cycling” model (Garlid et al., 1996, 2000) that originally postulated FA anion transport by UCP1 contradicted the electrophysiological data, because UCP1 lacks the ability to bind FA anions on the matrix side of the IMM, which is a requirement of the FA-cycling model (Fedorenko et al., 2012). Similarly, the H^+^-buffering model (Klingenberg and Huang, 1999; Klingenberg, 2017) could not explain all the new electrophysiological data because it did not take into account the FA anion transport by UCP1. Therefore, to explain all the available experimental data on UCP1, a new model of UCP1 operation was proposed (Fedorenko et al., 2012).

**FIGURE 5:**
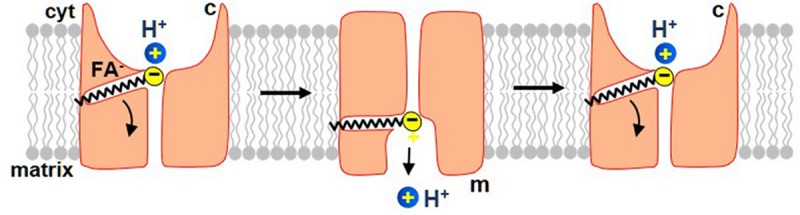
The proposed mechanism by which long-chain FA activate H^+^ current via UCP1. In this model, UCP1 cotransports one FA^–^ and one H^+^ per a transport cycle. The H^+^ and the FA^–^ bind to UCP1 on the cytosolic side of the IMM and, upon a conformational change, are translocated by UCP1 to the matrix side of the IMM. H^+^ is then released into the mitochondrial matrix, while the FA anion stays associated with UCP1 due to the hydrophobic interactions established by its carbon tail. Next, upon the second conformation change, UCP1 returns FA anion back to the cytosolic face of the IMM, where FA can initiate a new H^+^ translocation cycle. The translocation of FA anion to the cytosolic face of the IMM is assisted by the negative charge within the matrix. Also note that the charge translocation required for uncoupling occurs only in this step, while the previous step of FA^–^/H^+^ cotransport is electroneutral.

UCP1 belongs to the SLC25 family of mitochondrial carrier proteins that operate by an alternating-access mechanism of transport (Robinson and Kunji, 2006; Robinson et al., 2008; Kunji and Robinson, 2010). The new model of the FA-dependent H^+^ translocation by UCP1 assumes that UCP1 operates via a similar mechanism. Because UCP1 transports both FA anions and H^+^, the new model also postulates that UCP1 operates as a FA^–^/H^+^-cotransporter. However, because long-chain FA anions, the physiological activators of H^+^ leak via UCP1, cannot dissociate from UCP1, the translocation cycle results only in transmembrane transport of H^+^ (Figure 5). A single long-chain FA anion shuttles within UCP1 to enable transport of many H^+^ (Figure 5).

In this model, UCP1 has two conformation states. In one, the substrate-binding site (SBS) is exposed to the cytosolic side of the IMM (c-state), while in another it is exposed to the matrix side (the m-state). A long-chain FA anion and H^+^ bind to UCP1 in the c-state of UCP1. Upon a c-m conformational change the SBS becomes exposed to the matrix side of the IMM and H^+^ is released into the mitochondrial matrix. The long-chain FA^–^ remains anchored to UCP1 due to the hydrophobic interactions established. Next, the model postulates that while the long-chain FA anion is still associated with UCP1, the reverse conformational change is possible, which will return the FA anion back to the cytosolic face of the IMM to initiate another H^+^ transport cycle (Figure 5). This conformation change is likely assisted by the high negative voltage in the mitochondrial matrix that pushes the negatively charged FA anion toward the cytosolic face of the IMM. In this model, the FA^–^/H^+^ cotransport via UCP1 is electroneutral, and the charge translocation occurs only when the long-chain FA anion returns, after the release of H^+^, to the opposite side of the IMM (Figure 5).

Notably, the new model of UCP1 function is the simplest model that explains all of the electrophysiological data available. Future structure–function studies of UCP1 will provide specific data regarding the identity of FA anion and H^+^ binding sites within UCP1 and may lead to further refinement of the current FA^–^/H^+^ cotransporter model.

### FA Remove Purine Nucleotide Inhibition by Competition

Direct patch-clamp measurement of UCP1 currents also helped to address a controversy regarding the mechanism by which long-chain FA overcome inhibition of UCP1 by Mg^2+^-free purine nucleotides. Specifically, the indirect studies of H^+^ leak via UCP1 could not conclusively determine whether long-chain FA compete with purine nucleotides (Winkler and Klingenberg, 1994; Shabalina et al., 2004).

To inhibit UCP1, ATP^4–^ binds on the cytosolic side and occludes the UCP1 translocation pathway (Klingenberg, 2010, 2017). Direct electrophysiological analysis of UCP1 demonstrated that long-chain FA anions are UCP1 transport substrates that, similar to purine nucleotides, bind to the UCP1 translocation pathway only on the cytosolic side (Fedorenko et al., 2012). This result prompted re-evaluation of a competition between FA and purine nucleotides for binding to UCP1. Structurally, FA anions and ATP^4–^ are very different and unlikely to bind to the same site. However, the FA^–^ and ATP^4–^ binding sites may partially overlap or be located in immediate proximity, so that the electrostatic repulsion between the two negatively charged molecules results in competition.

**FIGURE 6:**
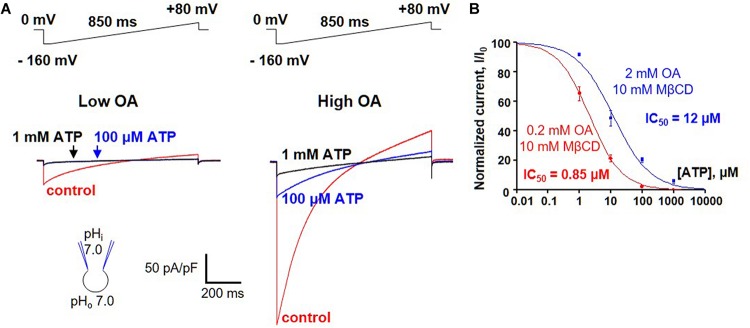
FA anions and purine nucleotides compete for binding to UCP1. **(A)** *Left panel:* representative H^+^ current via UCP1 induced by 0.2 mM OA/10mM MβCD in control (red), and in the presence of 100 μM (blue) and 1 mM (black) ATP. *Right panel:* representative H^+^ current via UCP1 induced by 2 mM OA/10 mM MβCD in control (red) and in the presence of 100 mM (blue) and 1 mM (black) ATP. **(B)** The dose-dependence of UCP1 inhibition by ATP at two different concentrations of activating OA. The H^+^ leak via UCP1 was activated either with 0.2 mM (red curve) or 2 mM OA (black curve) mixed with 10 mM MβCD. Error bars represent standard error of the mean (SEM). Reproduced with permission from Fedorenko et al. (2012).

A possibility of competition between long-chain FAs and purine nucleotides was addressed by measuring ATP^4–^ inhibition of H^+^ leak via UCP1 activated by two different concentrations of oleic acid (OA). Because brown fat IMM generates endogenous long-chain FA, OA was applied on a background of 10 mM methyl-β-cyclodextrin (MβCD) to limit the effect of these endogenous FA on UCP1 currents. The H^+^ current activated by 0.2 mM OA/10 mM MβCD was fully inhibited by 100 μM ATP, and subsequent addition of 1 mM ATP^4–^ caused no further inhibition (Figure 6A, left panel). In contrast, when 10-fold higher OA concentration (2 mM OA/10 mM MβCD) was used to activate H^+^ leak, it was inhibited by 100 μM ATP^4–^ only partially, and 1 mM ATP caused further inhibition (Figure 6A, right panel). ATP inhibited the H^+^ leak activated by 0.2 mM OA/10 mM MβCD with an IC_50_ of 2.1 ± 0.1 μM, whereas IC_50_ was 12.0 ± 2.8 μM in 2 mM OA/10 mM MβCD (Figure 6B). This IC_50_ shift demonstrates that long-chain FA anions compete with purine nucleotides for binding to UCP1 and are likely to remove purine nucleotide inhibition by competition.

What is the significance of purine nucleotide inhibition of UCP1? Basal levels of long-chain FA are likely to be present in the IMM even before adrenergic stimulation, and purine nucleotide can reduce the sensitivity of UCP1 to these long-chain FA to ensure that the UCP1-dependent H^+^ leak is not activated without a relevant physiological stimulus. Without purine nucleotides, even sub-micromolar long-chain FA can activate robust H^+^ leak via UCP1 (Fedorenko et al., 2012; Bertholet et al., 2017).

## Measuring Ucp1 Currents With Patch-Clamp Technique in Brown and Beige Fat Mitochondria

Here, we present a detailed protocol for measuring the H^+^ leak across the IMM of the thermogenic tissues brown and beige fat.

### Equipment and Solutions

#### Mitochondrial Patch-Clamp Set-Up

Please note that below we mention specific equipment used in our laboratory. We cannot exclude a possibility that other equipment may also be suitable for the purpose.

Olympus IX71 inverted microscope with differential interference contrast (DIC)
Olympus UPlanSApo 60X water immersion objective, numerical aperture 1.20
Vibration isolation table and Faraday cage
Axopatch 200B amplifier (Molecular Devices)
Digidata 1440AA Digitizer (Molecular Devices)
Micromanupulator MPC-385 (Sutter)
Bath reference electrode (3 M KCl-agar salt bridge)
Perfusion chamber with 0.13-mm glass coverslip bottom (Warner Instruments, RC-24E), connected to a gravity-fed perfusion system
Small round glass coverslips: 5 mm diameter, 0.1 mm thickness, cat. # 64-0731 (WPI Inc.).
0.1% gelatin (cat.# ES-006-B, Millipore)
MicroFil pipette filler (cat. # MF28G67-5, World Precision Instruments)
pClamp 10 (Molecular Devices) and Origin 9 (OriginLab) for data acquisition and analysis

#### Pipettes

Borosilicate glass capillaries with outer diameter of 1.5 mm, inner diameter of 0.86 mm, and an internal filament (Sutter Instruments, cat. # BF150-86-10)
Flaming/Brown Micropipette Puller, P97 (Sutter Instruments)
Micro Forge MF-830 for fire-polishing pipettes, equipped with Nikon MPlan 100/0.80 ELWD 210/0 (Narishige)

#### Mitochondrial Isolation

Potter-Elvehjem homogenizer, 10 ml capacity (Wheaton)
IKA Eurostar PWR CV S1 laboratory overhead stirrer
10 ml glass beakers and 35-mm plastic Petri dishes
French Press for disrupting mitochondrial outer membrane, for example, Thermo Electron French Press (FA-078A) with the Mini Cell (FA-003)
Refrigerated centrifuge with a relative centrifugal field (RCF) of up to 10,500 × g

#### Solutions for Mitochondrial Isolation and Patch-Clamp

Initial solution: 250 mM sucrose, 10 mM HEPES (4-(2-hydroxyethyl)-1-piperazineethanesulfonic acid), and 1 mM EGTA (ethylene-bis(oxyethylenenitrilo)tetraacetic acid, pH adjusted to 7.2 with TrisBase)
Hypertonic mannitol solution: 140 mM sucrose, 440 mM D-mannitol, 10 mM HEPES, and 1 mM EGTA (pH adjusted to 7.2 with TrisBase)
KCl storage solution: 750 mM KCl, 100 mM HEPES, and 1 mM EGTA (pH adjusted to 7.2 with TrisBase)
HEPES pipette solution: 130 mM TMA (tetramethylammonium hydroxide), 100 mM HEPES, 1 mM EGTA, 2 mM MgCl_2_ and pH 7.0 with D-gluconic acid (tonicity adjusted to ∼360 mmol per kg with sucrose)
MES pipette solution: 130 mM TMA, 100 mM MES (2-(N-morpholino)ethanesulfonic acid), 1 mM EGTA, 2 mM MgCl_2_ and pH 7.0 with D-gluconic acid (tonicity adjusted to ∼360 mmol per kg with sucrose)
KCl bath solution: 150 mM KCl, 10 mM HEPES, 1 mM EGTA and pH 7.0 with TrisBase (tonicity ∼300 mmol per kg)
HEPES bath solution: 150 mM HEPES, 1 mM EGTA and pH 7.0 with TrisBase (tonicity adjusted to 300 mmol per kg with sucrose)
MES bath solution: 150 mM MES, 1 mM EGTA and pH 7.0 with TrisBase (tonicity adjusted to 300 mmol per kg with sucrose)

#### Chemicals

CL 316.243 hydrate (Sigma-Aldrich Cat# C5976)
Guanosine 5′-diphosphate Tris salt (GDP, Sigma-Aldrich Cat# G7252)
Adenosine 5′-triphosphate disodium salt hydrate (ATP, Sigma-Aldrich Cat# A6419)
Oleic acid (Sigma-Aldrich Cat# O1008).
Arachidonic acid sodium salt (Sigma-Aldrich Cat# A8798, now Cat# SML1395)
Methyl-β-cyclodextrin (Sigma-Aldrich Cat# C4555)
1-Hexanesulfonate monohydrate sodium salt (Sigma-Aldrich Cat# 52862)
Arachidonic acid sulphonate sodium salt (Cayman Chemical Cat# 9001886)
1-Octadecanesulfonic acid sodium salt (TCI Chemicals Cat# O0124)

### Mitoplast Preparation From Brown and Beige Fat

#### Inducing Beige Fat in White Adipose Tissues

To induce beige adipocytes, mice are injected intraperitoneally with the selective β3-adrenergic receptor agonist CL316.243 (CL) at 1 mg/kg daily for 10 days (Bertholet et al., 2017), as also was reported previously (Himms-Hagen et al., 2000; Granneman et al., 2003, 2005). The induction of beige fat is discernible by distinctive morphological changes, with an increase in the number and a reduction in the size of cytoplasmic lipid droplets being the most obvious one (Figure 2). Mitochondrial biogenesis is another parameter used to identify “browning” of white fat (Figure 2), as detected by the increased expression of the transcriptional coactivator PGC1α (a key regulator of mitochondrial biogenesis) and mitochondrial proteins as COXIV and TOM20, in both inguinal and epididymal depots after CL treatment (Bertholet et al., 2017).

Our standard mitochondrial isolation protocol (see below) produces significant amounts of mitochondria from both inguinal and epididymal depots of CL-treated mice. In contrast, no mitochondria can be isolated from inguinal and epididymal fat of vehicle-treated mice (Bertholet et al., 2017). Therefore, all mitochondria isolated from white fat depots of CL-treated mice are newly formed beige fat mitochondria. These mitochondria are used in patch-clamp experiments to characterize UCP1 currents in beige fat.

#### Isolation of Mitochondria and Mitoplasts

Vesicles of isolated whole IMM, mitoplasts, are used for mitochondrial patch-clamp. The methodology for mitoplast preparation is based on: (1) isolation of mitochondria using differential centrifugation; (2) mechanical disruption of the outer mitochondrial membrane using a French press. The same protocol is used to isolate mitochondria/mitoplasts from both brown and beige fat. The French press procedure for mitoplast preparation (Decker and Greenawalt, 1977; Kinnally et al., 1993; Fedorenko et al., 2012) is preferred over the procedure using hypotonic swelling (Siemen et al., 1999; Szabo et al., 2005), because it has a potential to isolate IMM with fully preserved integrity, including the matrix and crista (Decker and Greenawalt, 1977). This is achieved by incubation of mitochondria in a hypertonic solution and using lower pressure during the French press procedure, so that the IMM does not expand above its normal volume while the outer membrane is removed (Decker and Greenawalt, 1977). In contrast, the standard hypotonic shock procedure that causes removal of the outer membrane always expands and stretches the IMM significantly, often causing IMM damage and matrix release. Mitoplasts generated by the osmotic swelling can be used for whole-IMM recording, but one can expect that the success rate will be lower.

We do not provide here a complete description of how to identify and dissect the fat pads. For more details about the dissection, please refer to previous dedicated articles (Mann et al., 2014). Here, we focus on how mitochondria can be isolated from dissected adipose tissues.

**FIGURE 7:**
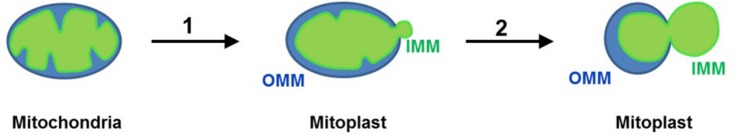
Preparation of mitoplasts (Leech and Holz, 1994). Mitochondria isolated from tissue lysate are subjected to low-pressure French press to rupture the OMM and release the IMM (mitoplasts are formed) (Suchyna et al., 2009); when mitoplasts are incubated in KCl solution, the IMM is further released from the OMM and mitoplasts assume an 8-shaped form. Remnants of the OMM are attached to the IMM. The IMM is shown in green, while the OMM is shown in blue.

– All solutions, glass homogenizers, pestles, and conical tubes must be ice-chilled.
– Euthanize the animal by CO_2_ asphyxiation followed by cervical dislocation. This method is consistent with the recommendations of the Panel on Euthanasia of the American Veterinary Medical Association and IACUC Committee.
– Isolate mouse interscapular brown fat, inguinal and/or epididymal adipose tissues. Clean brown fat from white fat. Remove from inguinal fat the lymphatic node and fibrotic tissues encapsulating the tissue.
– Transfer tissues in a beaker with 5 ml of “Initial” solution. Chop the tissue into thin, fine pieces using a micro-dissection scissor and transfer it to the glass homogenizer.
– Transfer tissue pieces to ice-chilled 10 ml Wheaton glass homogenizer (Teflon pestle). Attach the pestle of the homogenizer to the laboratory overhead stirrer and homogenize the diced tissue on ice with six slow strokes at 275 rot/min speed.
– Transfer the homogenized suspension to a 15 ml ice-cold conical tube and centrifuge it at 8,500 × g for 10 min at 4°C.
– Discard supernatant (containing the lipid phase).
– Resuspend the pellet in 5 ml of ice-cold “initial solution” and homogenize cells on ice with six slow strokes at 275 rot/min speed.
– Transfer homogenized suspension into 15 ml ice-cold conical tube and centrifuge at 700 × g for 10 min at 4°C.
– Transfer supernatant to a new ice-chilled tube and centrifuge at 8,500 × g for 10 min at 4°C to obtain a pellet of mitochondria.
– Discard supernatant.
– Resuspend the mitochondrial pellet in 3.8 ml of ice-cold “hypertonic-mannitol” solution and keep it on ice for 10–15 min.
– The steps of the procedure for mitoplast isolation are illustrated in Figure 7. To prepare mitoplasts, load the mitochondrial suspension into a 4 ml pre-chilled mini pressure cell (3/8” piston diameter) of the French press (Thermo Fisher). Set the French Press in the “Medium” mode and press the suspension through the pressure cell at 110 on the dial of the French press. The exit valve of the pressure cell should be set so that the suspension comes out of the cell at a rate of about 1 drop/s. Drops are collected in a 15 ml ice-chilled conical tube. This procedure results in rupture of the outer mitochondrial membrane. This method allows for a gentle and purely mechanical (no detergents are used) isolation of mitoplasts to preserve the integrity of the IMM (Decker and Greenawalt, 1977).

– Create pellet of mitoplasts by centrifugation at 10,500 × g for 10 min.
– Resuspend mitoplasts in 0.5–2 ml of ice-cold KCl storage solution, then transfer them to an ice-chilled conical tube and store the suspension on ice, protected from excess light. Mitoplasts are ready for patch-clamp recording immediately and will normally remain usable for about 3 h.

### Measuring UCP1 Currents Across the Whole Inner Mitochondrial Membrane

#### Pipettes and Solutions

The pipettes used for whole-mitoplast patch-clamp recordings are similar to those used for whole-cell recordings except the pipette tip is a smaller size.

We pull glass micropipettes from borosilicate glass filaments using a Sutter P97 micropipette puller. The pipettes are next fire-polished using a micro forge under 100X magnification (Nikon MPlan 100/0.80 ELWD 210/0 lens). The pipettes are prepared on the day of recording.

The pipette solution is filtered through a 0.22 μm filter. The pipette is back-filled with the pipette solution to 90% full using a 1 ml syringe connected to a MicroFil needle. The remaining bubbles are removed by gently tapping the pipette. Bath and pipette patch-clamp solutions are formulated to record H^+^ currents and contain only salts that dissociate into large anions and cations that are normally impermeant through ion channels or transporters. To record H^+^ currents via UCP1, the pipette is filled with HEPES pipette solution, while the bath contains HEPES bath solution. In contrast, to record FA anion currents via UCP1, we normally use MES pipette solution and MES bath solution. The pipette solution has about a 20% higher tonicity than the bath solution to prevent shrinking/collapse of the mitoplast after break-in or during patch-clamp recording. When filled with TMA-based pipette solution, the resistance of the pipettes should range between 25 and 35 MΩ.

#### Establishing the Whole-Mitoplast Mode and Measuring Mitoplast Membrane Capacitance

To reduce mitoplast adhesion coverslips are pre-incubated with 0.1% gelatin on the day of the experiment. Rinse 3–4 gelatin-coated coverslips with KCl bath solution and place in a well of a 4-well plate using a sharp curved forceps. Mix ∼35 μl of the concentrated mitoplast suspension with 500 μl of KCl bath solution in a 1.7 ml tube and plate on the top of coverslips. Incubate the plate on ice for 15–20 min, protected from light, for mitoplast sedimentation on the top of coverslips.

**FIGURE 8:**
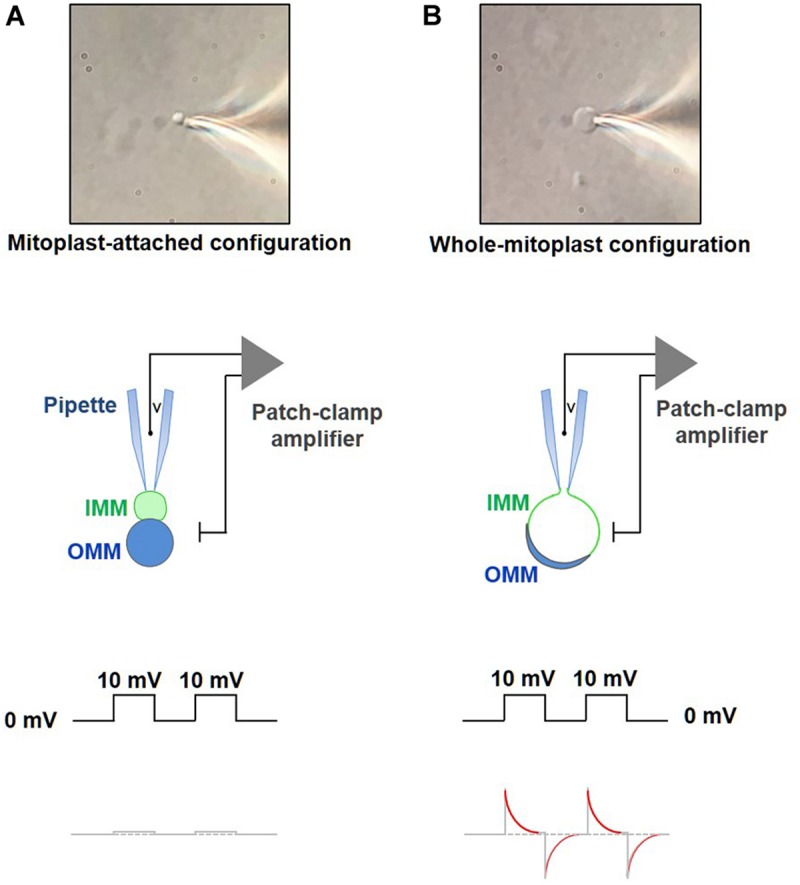
Formation of a gigaohm seal and breaking-in into a mitoplast. **(A)** Formation of the gigaohm seal between the glass patch pipette and the IMM (mitoplast-attached configuration). *Upper panel:* a photograph of a mitoplast attached to the glass patch-pipette in the mitoplast-attached configuration. *Middle panel:* a diagram illustrating seal formation between the patch pipette and the 8-shaped mitoplast. The pipette is attached to the IMM lobe (green). When observed with a DIC optics, the IMM lobe is less optically dense as compared to the lobe that contains both, IMM and OMM (blue). *Lower panel:* the current response to square 10-mV voltage pulses in the mitoplast-attached configuration after compensation of stray capacitance transients. **(B)** Formation of the whole-mitoplast (whole-IMM) configuration. Establishing the mitoplast-attached configuration is followed by break-in into the mitoplast to achieve a whole-mitoplast configuration for recording currents across the whole IMM. The IMM patch under the pipette is destroyed by high-amplitude voltage pulses (200–500 mV). *Upper panel:* a photograph of a mitoplast attached to the glass patch-pipette after breaking-in and formation of the whole-mitoplast configuration. Note a change in the shape of the mitoplast as compared to that in **(A)**. *Middle panel:* a diagram illustrating the patch-pipette and the mitoplast after formation of the whole-mitoplast configuration. After the break-in, the interior of the mitoplast is perfused with the pipette solution. As the result, the IMM swells and is completely released from the OMM, while the mitoplast assumes a round shape. *Lower panel:* the current response to square 10 mV voltage pulses in the whole-mitoplast configuration. Note appearance of current transients associated with the capacitance of the whole IMM. Red color in the current response represents a single exponential fit to the decay phase of the capacitance transient.

– Fill the bath chamber with KCl bath solution and position one of the coverslips with mitoplasts in the middle of the chamber. During this time, switch the perfusion off to prevent the mitoplasts from being washed away.
– Scan the coverslip using a 60X objective to check the quality of the preparation (the best quality being with few debris and distinct mitoplasts). To succeed in the mitoplast-attached step, it is important to choose an individual free-floating 8-shaped mitoplast. One should avoid round-shaped mitoplasts in which the IMM is completely released from the OMM, because integrity of such mitoplasts is often compromised. Engagement of the additional 1.6X magnification of an IX71 microscope helps with the selection of an 8-shaped mitoplast with a clean surface. The 8-shaped form of the mitoplast is due to protrusion of the inner membrane through a hole in the outer membrane caused by the French press treatment (Figure 7). An 8-shaped mitoplast has two lobes. The lobe of a lower optical density contains only the IMM released from the OMM, while the more optically dense lobe contains the OMM with a fraction of the IMM still inside it. Using quality optics as described above, the less dense lobe containing only the IMM can be easily identified.
– Fill the pipette with a pipette solution and load into the pipette holder. Using the micromanipulator, bring the pipette into the bath solution and further down, so that it is located just above the selected mitoplast and is visible in the field of view. Slight positive pressure must be applied into the pipette to prevent bath solution containing membrane debris from entering into the pipette. Set the amplifier to voltage-clamp mode and zero the pipette offset. In the seal test, we usually apply a 10 mV test pulse at 33 Hz from a holding potential of 0 mV.
– Switch the micromanipulator to a fine movement mode and slowly approach the less dense lobe, i.e., IMM) of the selected mitoplast.
– When the mitoplast fluctuating near the bottom of the coverslip presents its IMM lobe toward the pipette, quickly apply a gentle negative pressure to form a gigaseal (>2–5 GΩ, Figure 8A). To facilitate the formation of the gigaseal, a small negative pressure can be applied into the pipette. If the gigaseal is not formed, and formation of the mitoplast-attached configuration is attempted with a new pipette and a new mitoplast.

– After the mitoplast-attached mode is formed, gently lift the mitoplast from the coverslip to prevent disruption of the seal due to pipette drift during the experiment.
– The stray capacitance transients should now be maximally compensated, before achievement of the whole-mitoplast configuration. This is a very important step to achieve a correct measurement of the mitoplast membrane capacitance (Cm) after the break-in.
– Apply single short-duration (5–15 ms) voltage pulses (250–600 mV) to break-in into the mitoplast and achieve the whole-mitoplast configuration. Normally, the longest duration and the lowest voltage are used first. Break-in is attempted several times (normally five times) with a specific combination of voltage and duration. If the break-in is not achieved, voltage of the break-in step is gradually increased (in ∼50 mV increments), while its duration is gradually decreased. To break-in, voltage pulses can be combined with a light suction. The break-in voltage pulse protocol is created using the high-voltage command input on the back of the Axopatch 200B. The process of break-in is monitored using the Membrane Test tool of pClamp. A successful break-in results in the reappearance of capacitance transients (Figure 8B) with almost no steady-state current (i.e., non-specific leak current). To calculate the electrode access resistance (Ra) and the membrane capacitance (Cm), the Membrane Test fits the capacitance current transients with an exponential function (Figure 8B). After the break-in, Ra should be between 40 and 80 MΩ. Brown and beige fat mitoplasts (2–6 μm in size) used for patch-clamp experiments typically have membrane capacitances of 0.5–1.1 pF. Upon break-in, the mitoplast usually swells due to the higher tonicity of the pipette solution and becomes round and more transparent (after the optically dense matrix content diffuses into the pipette) with remnants of the outer membrane sometimes visible on the side (Figure 8B).

#### Measuring H^+^ Leak via UCP1

To measure the H^+^ current via UCP1, we regularly use a HEPES pipette solution and HEPES bath solution, both at pH 7.0 (Fedorenko et al., 2012; Bertholet et al., 2017). These bath and pipette solutions are formulated to record H^+^ currents and contain only salts that dissociate into large anions and cations that are normally impermeant through ion channels or transporters. In these recording solutions, the H^+^ current via UCP1 can be observed without addition of exogenous FA. This is because, as mentioned above, the IMM of brown and beige fat contain endogenous FA generated by an IMM-associated phospholipase activity (Fedorenko et al., 2012; Bertholet et al., 2017). The H^+^ current via UCP1 that is activated by the endogenous membrane long-chain FA is pH-dependent. It is largest with a symmetrical (the same in the bath and pipette solutions) alkaline pH ∼9.0 and is dramatically reduced at symmetrical pH 5.0 (Fedorenko et al., 2012). This pH dependence is likely to be due to both the pH-dependence of the FA-associated UCP1 and the pH-dependence of the IMM-associated phospholipase(s) responsible for the production of the endogenous FA (Fedorenko et al., 2012). It is most convenient to record the H^+^ current via UCP1 at symmetrical pH 7.0, because these conditions are close to the physiological environment, the amplitude of the H^+^ current via UCP1 is large, and the IMM is stable. In contrast, at both extremes, pH 5.0 and pH 9.0, the integrity of the IMM is severely compromised.

To record H^+^ currents via UCP1:

– Immediately after a successful break-in, replace the KCl bath solution with the HEPES bath solution.
– Apply a 850 ms ramp protocol from −160 mV to +100 mV with a 5 s interval, while holding the mitoplast at 0 mV (Figure 3). This voltage protocol covers the whole range of physiological potentials the IMM is likely exposed to as well as some positive voltages that are not physiological but can help the biophysical analysis of UCP1 currents.
– Application of the ramp protocol elicits a large-amplitude H^+^ current across the IMM of brown and inguinal beige fat (Fedorenko et al., 2012; Bertholet et al., 2017). In contrast, only 15% of epididymal beige fat mitoplasts develop a UCP1-dependent H^+^ current (Fedorenko et al., 2012; Bertholet et al., 2017).
– When the H^+^ current develops in response to the ramp protocol, wait for stabilization of the current amplitude.
– For quantification of the amplitude of the H^+^ current via UCP1, it is crucial to determine the baseline corresponding to zero UCP1 current. To achieve this, perfuse the bath with either the UCP1 inhibitor GDP (1 mM) or a FA chelator (such as 0.5% FA-free bovine serum albumin or 10 mM MβCD that extract endogenous membrane FA). The H^+^ current via UCP1 will be fully inhibited, and the remaining current will be the baseline current from which the amplitude of UCP1 currents is to be measured. UCP1 currents can be further normalized per mitoplast capacitance (Cm) to obtain current density (pA/pF), which facilitates comparison of UCP1 current amplitudes in different mitoplasts.
– After extraction of endogenous FA with MβCD/albumin and deactivation of the H^+^ current via UCP1, the current can be reactivated by addition of exogenous arachidonic acid or OA (1 μM or similar concentrations) (Fedorenko et al., 2012; Bertholet et al., 2017). Alternatively, after depletion of endogenous FA from the IMM, MβCD/albumin can be washed out from the bath, and endogenous membrane FA will be partially regenerated by the phospholipases within a few minutes, reactivating the H^+^ current via UCP1 (Fedorenko et al., 2012; Bertholet et al., 2017).
– To compare H^+^ transport activities of UCP1 in individual mitoplasts (especially mitoplasts from different tissues such as brown and beige fat), it is important to eliminate the effect of endogenous FA (which can be present at different concentrations, especially in mitoplasts from different tissues) and activate UCP1 currents with the same concentration of exogenous FA (Fedorenko et al., 2012; Bertholet et al., 2017). To achieve this, apply the HEPES bath solution containing 10 mM MβCD to extract endogenous FA and re-activate the UCP1 current by exogenous FA on the background of MβCD (for example, 10 mM MβCD mixed with 0.5 mM OA). Application of exogenous FA on the background of MβCD ensures that the endogenous FA generated within the IMM are continuously eliminated and do not affect the measurements of the H^+^ current via UCP1.
– Clamping the concentration of activating FA at certain levels can be important not only for comparing UCP1 activity in mitochondria of different tissues, but also for studying the mechanism of UCP1 interaction with purine nucleotides. In particular, competition of purine nucleotide and FA for binding to UCP1 can be studied by comparing UCP1 inhibition by a purine nucleotide (e.g., Mg^2+^-free ATP) at two 10-fold different FA concentrations (Figure 6). To eliminate the effect of endogenous membrane FA and achieve precise control over the concentration of activating FA, the H^+^ current is activated with exogenous OA (e.g., 0.2 and 2 mM) applied on a background of 10 mM MβCD.

#### Measuring Fatty Acid Anion Currents via UCP1

To measure FA anion currents via UCP1, UCP1 must be activated by low-pKa FA analogs. Because transport of H^+^ by UCP1 depends on H^+^ binding to the activating FA, low-pKa FA analogs that cannot bind H^+^ at physiological conditions do not activate H^+^ currents (Fedorenko et al., 2012; Bertholet et al., 2017). Instead, they reveal FA anion transport by UCP1 (Fedorenko et al., 2012; Bertholet et al., 2017). In contrast to measuring the H^+^ current via UCP1, FA anion currents are best measured at symmetrical pH 6.0 using MES bath and MES pipette solutions. In symmetrical pH 6.0, the activity of the IMM-associated phospholipases that generate endogenous FA is suppressed and specific activation of UCP1 currents by exogenous low-pKa FA analogs can be achieved (Fedorenko et al., 2012; Bertholet et al., 2017).

To record FA anion currents via UCP1:

– Immediately after a successful break-in, replace the KCl bath solution with the MES bath solution containing 10 mM MβCD to extract endogenous FA from the IMM.
– Apply a voltage step protocol (+50 mV, −50 mV, +50 mV, Figure 4) from a holding potential of 0 mV. Record the baseline current.
– Apply desired concentration of a low-pKa FA analog. The binding affinity of FA and their ability to induce FA anion currents via UCP1 depend on the analog’s hydrophobicity and the number of carbons in the hydrophobic chain. For long-chain low-pKa FA analogs, use low micromolar concentrations, but millimolar concentrations of medium and short-chain low-pKa FA analogs are required to activate the FA anion current via UCP1 (Fedorenko et al., 2012).
– Similar to the H^+^ currents, FA anion currents via UCP1 can be inhibited by application of 1 mM GDP or another Mg^2+^-free purine nucleotide to the bath solution.

## Discussion

The whole-IMM patch-clamp for the first time allowed high-resolution functional analysis of UCP1 in the native membrane environment. It allows precise control of critical experimental conditions, such as pH, membrane potential, and concentration of different ions and metabolites across the IMM. It measures transmembrane currents with very high time (<1 ms) and amplitude (<1 pA) resolution and is the only method by which the ionic selectivity of the current can be reliably determined. This method was successfully used to identify and characterize the H^+^ leak mediated by UCP1 in brown fat mitochondria and to shed light on the mechanisms of mitochondrial thermogenesis in beige fat. Combined with classical methods, such as mitochondrial respirometry, it elevates the study of the mechanisms of mitochondrial uncoupling and thermogenesis to a previously unattainable level, facilitating studies that will rigorously address some of the most long-standing questions in the field of bioenergetics.

## Data Availability Statement

The raw data supporting the conclusions of this article will be made available by the authors, without undue reservation, to any qualified researcher.

## Ethics Statement

All animal experiments were performed according to procedures approved by the UCSF Institutional Animal Care and Use Committee.

## Author Contributions

AB and YK conceived and wrote the manuscript.

## Conflict of Interest

YK was a co-founder of Equator Therapeutics.

The remaining author declares that the research was conducted in the absence of any commercial or financial relationships that could be construed as a potential conflict of interest.

## Abbreviations

IMM: Inner Mitochondrial Membrane
OMM: Outer Mitochondrial Membrane
UCP: Uncoupling Protein
UCP1: Uncoupling Protein 1
FA-: Fatty Acid Anion
ETC: Electron Transport Chain
FA: Fatty Acids
SBS: Substrate Binding Site
TMA: Tetramethylammonium Hydroxide
GDP: Guanosine 5′-diphosphate
ATP: Adenosine 5′-triphosphate
CL: CL 316.243
Ra: Electrode Access Resistance
Rm: Membrane Resistance
Cm: Membrane Capacitance
M β CD: Methyl- β -cyclodextrin.
